# Triage processes at multidisciplinary chronic pain clinics: An international review of current procedures

**DOI:** 10.1080/24740527.2017.1331115

**Published:** 2017-10-23

**Authors:** M. Gabrielle Pagé, Daniel Ziemianski, Yoram Shir

**Affiliations:** a Alan Edwards Pain Management Unit, Montreal General Hospital, Montreal, Quebec, Canada; b Centre de recherche du Centre hospitalier de l’Université de Montréal (CRCHUM), Montreal, Quebec, Canada; c School of Physical and Occupational Therapy, McGill University, Montreal, Quebec, Canada; d Alan Edwards Center for Research on Pain, Genome Building, McGill University, Montreal, Quebec, Canada; e Department of Anesthesia, McGill University, MUHC, Montreal, Quebec, Canada

**Keywords:** triage, multidisciplinary pain clinic, chronic pain, best practice guidelines, referral

## Abstract

**Background**: Multidisciplinary pain clinics are considered the gold standard for the treatment of chronic pain, yet access to such clinics is difficult and patients’ conditions deteriorate while waiting. Instituting a triage process is one way of reducing wait time for some patients and ensuring optimal access given the limited resources available. Surprisingly, there are no established guidelines on how to optimally triage chronic pain patients at tertiary multidisciplinary pain clinics.

**Aims**: The goal of this study was to gather information regarding existing triage systems in multidisciplinary chronic pain clinics worldwide as an initial step toward establishing a definitive evidence-based set of triage guidelines.

**Methods**: A total of 66 multidisciplinary pain clinics worldwide completed an online survey detailing current triage practices at their clinic. The survey was distributed via international and national pain associations.

**Results**: Results showed that the vast majority of multidisciplinary pain clinics (94%) use a triage system, yet many difficulties with these systems have been identified (time requirement, administrative burden, lack of control over scheduling, missing high-priority patients, and prioritizing low-priority patients). The level of satisfaction was noted to be higher in those clinics using a structured triage template.

**Conclusions**: This study identified a need for the elaboration of best practice clinical guidelines for triage processes at tertiary pain clinics. The use of a structured referral template could become a central element to such guidelines.

## RéSUMé

**Contexte**: Les cliniques multidisciplinaires de la douleur sont considérées comme l’exemple idéal pour le traitement de la douleur chronique. L’accès à ces cliniques est toutefois difficile, de sorte que l’état de santé des patients se détériore pendant la période d’attente. L’instauration d’un processus de triage est l’une des façons de réduire le temps d’attente pour certains patients et d’assurer un accès optimal aux ressources limitées qui sont disponibles.

**Objectifs**: Le but de cette étude était de recueillir de l’information concernant les systèmes de triage existants dans les cliniques multidisciplinaires de la douleur à travers le monde, comme première étape vers l’établissement de lignes directrices définitives fondées sur des données probantes.

**Méthodes**: Au total, 66 cliniques multidisciplinaires de la douleur à travers le monde ont répondu à un sondage en ligne portant sur les pratiques de triage dans leur clinique. Le sondage a été distribué par l’entremise d’associations nationales et internationales de la douleur.

**Résultats**: Les résultats ont démontré que la vaste majorité des cliniques multidisciplinaires de la douleur (94 %) utilisent un système de triage. Toutefois, de nombreuses difficultés ont été recensées en lien avec ces systèmes (temps nécessaire, fardeau administratif, manque de contrôle sur la prise de rendez-vous, difficulté à identifier certains patients prioritaires et priorisation des patients non prioritaires). Le niveau de satisfaction était plus élevé dans les cliniques qui utilisaient un modèle de référence structuré pour le triage.

**Conclusions**: Cette étude a mis en évidence la nécessité d’élaborer des lignes directrices cliniques indiquant les pratiques exemplaires pour les processus de triage dans les cliniques de la douleur de niveau tertiaire. L’utilisation d’un modèle de référence structuré pourrait être un élément central de ces lignes directrices.

## Introduction

Based on North American, European and Australian studies, chronic pain affects approximately one fifth to one quarter of the population,^1–4^ yet there are many difficulties accessing good quality health care for its treatment. At the primary care level, it has been documented that family physicians are poorly equipped in terms of knowledge and resources to satisfactorily address the needs of chronic pain patients (inadequate training in pain management, time constraints, lack of remuneration).^5,6^ This is reflected in the research findings from the province of Ontario, Canada, where primary care practitioners prefer to refer chronic pain patients to specialized centers (university-affiliated hospital-based pain clinics), and many of those practitioners refer their chronic pain patients exclusively to tertiary-care level clinics.^7^ Multidisciplinary pain clinics, often associated with tertiary care levels, are considered the gold standard for the treatment of chronic non-cancer pain.^8–13^ Multidisciplinary pain clinics are dedicated to the treatment of pain, composed of professionals from multiple disciplines (e.g., medicine, nursing, mental health, physical therapy) who collaborate to provide evaluation and optimal management of pain.^14^ In Canada, the median time to access such specialized services, however, can be as long as 2 years in certain regions and is less than 6 months in fewer than half of clinics.^15^ Similar findings have been obtained in other countries. For example, the wait time for chronic pain treatment in teaching hospital pain clinics and general hospital pain clinics in Scotland is more than 3 months and 9 weeks, respectively, for more than half of patients.^16^ Results for the Canadian Pain Society Wait Times Task Force have shown that approximate waits vary between 3 weeks to up to 5 years depending on the country and type of pain clinic.^17^ According to a study conducted in Canada, two thirds of patients on waitlist experience severe levels of pain (7 or more out of 10), half experience moderate to severe levels of depression, and three quarters report that their pain interferes with normal work duties.^18^ While on a waitlist, patients experience a worsening of their chronic pain condition and associated symptoms, including depression and quality of life.^19^ The costs (in 2010) associated with being on a waitlist have been estimated to be CAD $1462 monthly.^20^

Little guidance exists on optimal ways to manage long waiting lists for chronic pain treatment.^21^ A triage process, whether based on pain severity, risk of deterioration, level of psychological distress, work status, likelihood of treatment response, is one way of reducing wait time for some priority patients and ensuring optimal access given the limited resources available. Effective triage processes would allow the identification of patients who need to be prioritized based on the desired outcome (e.g., likelihood of treatment success) or on patient characteristics (e.g., work status, psychological distress). Surprisingly, there are no established guidelines on how to optimally triage chronic pain patients at tertiary multidisciplinary pain clinics.^17^ Minimal data on triage processes exist for orthopedic spine clinics.^22,23^ A survey of Australian chronic pain management services found that 83% of outpatient care clinics had a triage process for newly referred patients to identify urgent cases of cancer pain, acute neuropathic pain, and high levels of distress; those identified as urgent had wait times of less than 4 weeks.^24^

The goal of this study was to gather information regarding existing triage systems in multidisciplinary chronic pain clinics worldwide as an initial step toward establishing a definitive evidence-based set of triage guidelines.

## Materials and methods

### Participants

Targeted participants were directors, administrators, or employees of multidisciplinary pain clinics. The survey was open between October and December 2015. To be considered a multidisciplinary clinic, three or more different disciplines of health professionals must have been involved in providing care at the clinic. Tertiary care clinics (private/public and in-hospital, outside of hospital, and university affiliated) were included.

### Procedure

The study was approved by the McGill University Health Center Research Institute Research Ethics Board. Pain clinics were contacted indirectly through national pain organizations, which were asked to distribute the survey to their members via e-mail. Electronic invitations included a summary of the survey description and purpose, consent information, and a link to access the online survey hosted on LimeSurvey (www.limesurvey.org). Surveys were to be completed by members of pain clinics; such members could be the director, administrator, or an employee.

First, two international organizations were contacted to distribute the survey invitation letter to their members, but neither agreed. As such, a country-specific approach was adopted. Health care systems and referral processes of North American and European countries were examined and countries to approach for study participation were selected if (1) the structure of their health care system was similar to that of Canada and (2) a referral is needed to access multidisciplinary pain clinics (tertiary care). As such, countries such as the United States (health care system very different from Canada) and England (no referral needed to access multidisciplinary pain treatment) were excluded. When available, lists of service providers across countries were consulted (e.g., Canada, Israel). A total of nine countries were identified and their national pain organizations approached. Among the nine countries approached (Canada, Australia, New Zealand, Iceland, Sweden, Finland, Denmark, Norway, and Israel), five national pain organizations agreed to participate and sent an e-mail containing the survey invitation letter to all of their members. One of these organizations also agreed to send a reminder e-mail about the survey to their participants a few weeks later. Organizations that did not answer our initial request were contacted a second time.

### Measures

The survey consisted of 115 items (see Appendix). The first four questions collected information about the respondent’s location, clinic setting (in-hospital, outside of hospital, university affiliated, private clinic, other), and clinic staffing (profession/role of respondent and whether respondent was clinic director) using multiple-choice and multiple-answer formats. Respondents were asked to indicate the volume of newly referred patients, the number of new patients seen on an annual basis, and the average wait time using three interval-scale, multiple-choice questions. Respondents were asked to rank criteria used to triage new patients for appointments (referral, date, etiology of pain, type of pain, pain level, past pain therapies, psychosocial status, age, work status, insurance/benefits status) and then to indicate any additional procedures used to triage patients (direct communication with referring clinician or family physician, structured referral template, institution-wide system for scheduling patients). Respondents who indicated the use of a structured referral template were asked to describe it using an open-ended question. Respondents were then asked to identify the various professionals involved in the triage process and to indicate their satisfaction with their clinic’s triage process using a five-point Likert scale (1 = *very unsatisfied*; 5 = *very satisfied*). They were then asked to indicate weaknesses of the triage process using a multiple-answer (missing high-priority patients, prioritizing low-priority patients, time required, administrative burden, lack of control over scheduling) and an open-ended question. Finally, respondents were provided the opportunity to write any comments or additional feedback in an open-ended long text response.

### Data analysis

Survey data were primarily analyzed using descriptive statistics (frequency, means, Spearman correlation) to examine answers to the different survey questions and provide an overview of existing triage processes across various multidisciplinary pain settings. Binary logistic regression analysis using a forward conditional approach was used to identify predictors (number of triage criteria used, use of structured referral template, direct communication with family physician, direct communication with referring physician, institution-wide scheduling system, use of questionnaire data or medical records) of satisfaction with the triage process (satisfied/very satisfied vs. neutral/dissatisfied/very dissatisfied).

## Results

A total of 72 pain clinics completed the survey; three entries were excluded because they originated from the same pain clinic and another three pain clinics were excluded because they reported having fewer than three professional disciplines working within the clinic. As such, a total of 66 multidisciplinary pain clinics from five different countries (Australia, Canada, Israel, New Zealand, Sweden) completed the survey and were included for data analysis (see Table 1). Most pain clinics were public (80%) and located at a hospital (60%); 17% were affiliated with a university. The number of different pain specialties ranged from three to 15 with a median of six types of specialists per clinic.

**Table 1.** Descriptive statistics of participating pain clinics.

Variables	*N*	%
**Locations of pain clinics**		
Australia	44	66.7
Canada	11	16.7
Israel	5	7.6
New Zealand	3	4.5
Sweden	2	3.0
Unspecified	1	1.5
**Type of settings**		
Public	53	80.3
Private	13	19.7
Hospital clinics	39	59.1
Outside hospital clinics	16	24.2
University-affiliated clinics (in or outside of hospital)	11	16.7
**Number of different pain specialists per clinic**		
Mean ± SD	6.9 ± 2.8	
Median (range)	6 (3–15)	
Three to five different types of professionals	22	33.3
Six to seven different types of professionals	22	33.3
Eight to ten different types of professionals	16	24.3
More than ten different types of professionals	6	9.1

### Composition of multidisciplinary pain clinics

**Figure 1.**
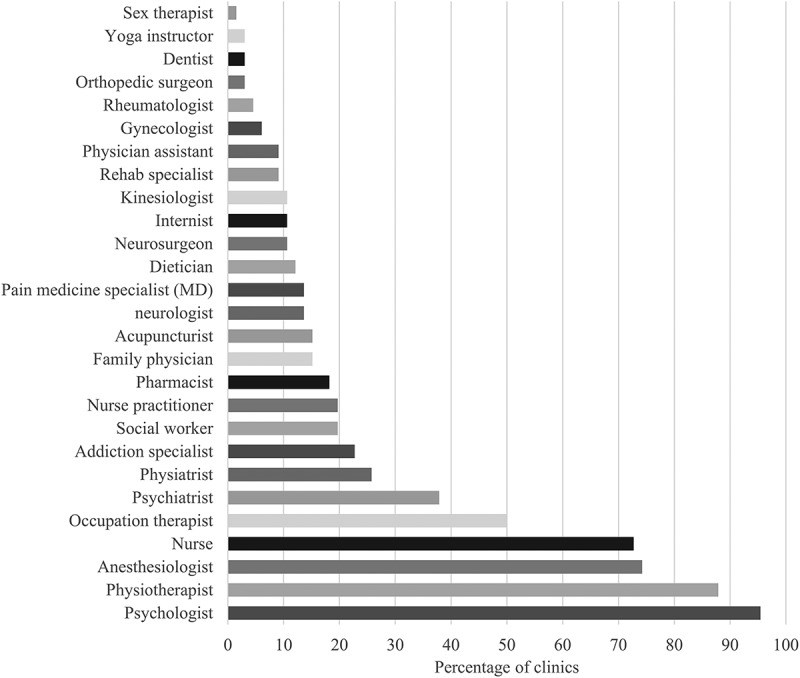
Percentage of multidisciplinary pain clinics reporting working with at least one team member of this profession.

As shown in Figure 1, ≥50% of pain clinics reported working with team members from the following professions: psychology (95%), physiotherapy (88%), anesthesiology (74%), nursing (73%) and occupational therapy (50%). Physiatrists (38%) and psychiatrists (26%) were also present in more than one quarter of pain clinics.

### Number of referrals, new patients, and wait times

**Figure 2.**
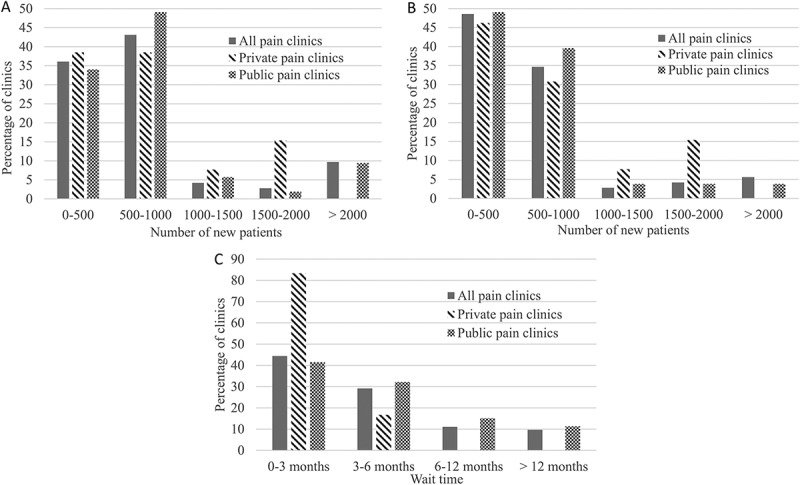
(A) Average number of new referrals yearly reported by public and private clinics. (B) Average number of new patients seen yearly by public and private pain clinics. (C) Average wait time for the first visit at the pain clinic.

The majority of clinics (82%) received fewer than 1000 new referrals yearly (see Figure 2) and 10% of clinics reported more than 2000 new referrals per year. Similarly, most clinics (86.4%) saw fewer than 1000 new patients yearly. Nearly half of clinics (49.2%) reported their average wait time to be less than 3 months and close to one third of clinics reported an average wait time between 3 and 6 months. Though private and public clinics reported no significant differences in terms of number of referrals or number of new patients seen (*P* > 0.05), private clinics all reported average wait times of less than 6 months and average wait times varied between less than 3 months and more than 12 months for public clinics (see Figure 2).

Spearman correlation (*r* = 0.829) showed a significant correlation between reported number of new patients referrals and new patients seen at the pain clinic (*P* < 0.001). Also significant was the correlation between average wait time and number of new patient referrals (*r* = 0.419; *P =* 0.001) as well as that between average wait time and number of new patients seen at the pain clinic (*r* = 0.246; *P =* 0.048).

### Triage processes

Ninety-four percent of clinics reported using a triage system; the remaining 6% reported using only the referral date as the triage criterion. The majority of clinics (56%) reported using eight or nine criteria for triage of new patients and approximately one third of clinics reported using three or fewer criteria.

**Figure 3.**
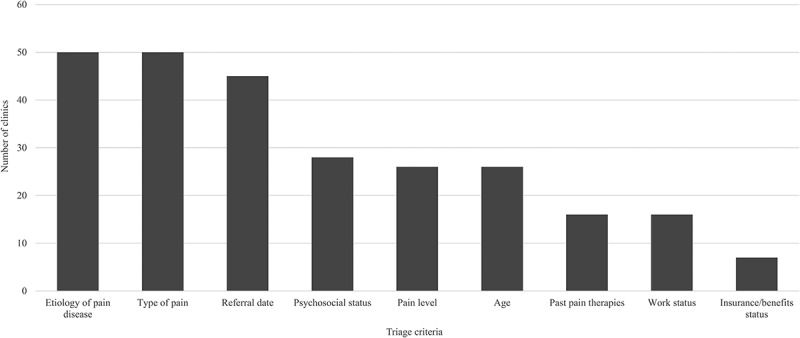
Number of multidisciplinary pain clinics who identified these criteria as part of their top five triage criteria.

The most important criterion for triage was etiology of pain disease for 41% of clinics, referral date for 26%, and type of pain for 15%. Figure 3 shows the criteria most consistently identified as the top five triage criteria by pain clinics. These include type of pain, etiology of pain disease, referral date, psychosocial status, and pain level.

**Figure 4.**
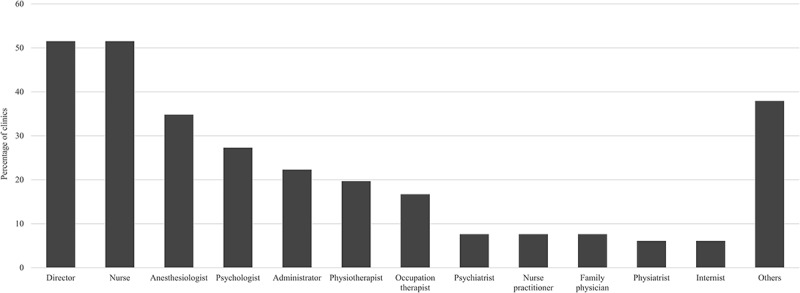
Professionals involved in the triage process.

In addition to the above-described triage criteria, the following procedures used for triage were reported by different pain clinics: direct communication with referring clinician (65.2%), direct communication with family physician (47.0%), requiring the use of a structured referral template (43.9%), the use of an institution-wide system for scheduling patients (24.2%), direct contact with patients (or parents; 8.2%), and the use of questionnaire data or medical records (11.5%). More than half of pain clinics reported relying on the clinic director and nurse for triage. Other professionals involved in the triage process are shown in Figure 4. The different comments on triage procedures provided in the surveys are reported in Table 2; among them, some clinics reported having implemented a pain management program for all patients before the triage process, prioritizing specific pain conditions (e.g., cancer-related pain, complex regional pain syndrome) or cultural factors (e.g., closing the gap measures).

**Table 2.** Additional information provided by participants regarding triage procedures, triage process weaknesses, and referral templates.^a^

What additional procedures are being used to triage patients?	
Cultural factors	*We have CULTURAL STATUS which differs from psychosocial. Children who identify as being of Aboriginal, Torres Strait Islander or refugee origin are prioritised—target is to see within 90 days. This is a “Closing the Gap” measure.*
Education and orientation seminars	*80% of patients referred are invited to attend an early group Education and Orientation seminar. The initial triage screens out patients unsuitable for the group seminar. Using the early group intervention has simplified the triage process and improved patient flow.*
	*Have streamlined triage process better e.g., referral letter only will pre-empt an Understanding Pain education session and they then bring referral Questionnaire if they wish to proceed.*
	*We have implemented the STEPS (self-training educative program) model … in which all patients are offered attendance at a short (about 12 hours over 2 days) educational program about chronic pain, and with moderate emphasis on the role of self-management: therefore we pretend to minimise the role of triage.*
Specific pain characteristics	Patients are excluded if: Current addiction Incomplete referral information Previously seen by pain specialists Clinic has nothing else to offer
	Prioritized patients: Cancer pain Acute pain Complex Regional Pain Syndrome (CRPS) Shingles Trigeminal neuralgia Phantom limb Suicidality Ability to work is at risk (especially in patients less than 50 years old) Condition possibly responsive to procedures (e.g., radiculopathic pain, cervical facet pain)
Helping in patient management while on waitlist	*The wait to MD assessment can be >12 months, but the wait to begin work with the team is negligible. MD assessments are booked in the order that referrals are received, but the triage nurse reviews a questionnaire submitted by the patient to determine if we can help the referring MD with a telephone consult in the meantime, so that medical management can be optimized right away.*
Other weaknesses to triage process	
Referral volume	*Due to the big number of referrals there is a minimal triage.*
	*Work load may mean we do not follow up referrals. We do send unable to contact letters.*
Lack of empirical evidence	*Lack of evidence based criteria to support and validate current prioritizing methods of triage.*
	*The lack of transparency in triage process is most frustrating.*
	*A standardised triage system that is fair and consistent would be a bonus and make the entry criteria and wait time more transparent.*
Referral information	*Extremely poor information by referring MD.*
	*Referral information not read properly.*
Team cohesion	*Team dialog needs improvement.*
Pain education process	*… the paradigm of “open entry” to STEPS* [pain education prior to triaging] *fails in patients with language and cognitive barriers: we struggle to acquire adequate information for triage and to see such patients in a timely fashion.*
Structured referral template	
Example 1	Demographics, pain, and related problems experienced (sleep, psychological, emergency consultation or hospitalization, etc.), relevant clinical, surgical, imaging history, treatments, medications Available online: www.aci.health.nsw.gov.au/__data/assets/pdf_file/0005/212774/NSW_HEALTH_Referral_Guide_to_Adult_and_Paediatric_Chronic_Pain_Services_interactive_070414_i.pdf?SQ_DESIGN_NAME=text
Example 2	Pain registry—Electronic Outcomes Pain Collaboration (e.g., Brief Pain Inventory (BPI), Depression Anxiety Stress Scales (DASS), Pain Catastrophizing Scale (PCS), Pain Self-Efficacy Questionnaire (PSEQ), work status, pain diagram)
	Questionnaire data used as triage information by some clinics. http://ahsri.uow.edu.au/eppoc/index.html
Example 3	Pain descriptive and diagnosis, date of onset, brief history, comorbidities, previous treatments received, physician assessment of triage level, relevant reports from previous investigations (50% compliance in providing this information)
Example 4	Referral questionnaire and patient questionnaires (BPI, PSEQ, Kessler Psychological Distress Scale (K10), analgesic effectiveness, substance use)
Example 5	General practitioner and specialist referral information, pain condition, medical history, pain medications, past treatment, Opioid monitoring registration, health care utilization, standardized patient questionnaires (BPI, PSEQ, K10), and review of mental health database

*Note*. ^a^Italics indicate direct quotations from participants.

**Figure 5.**
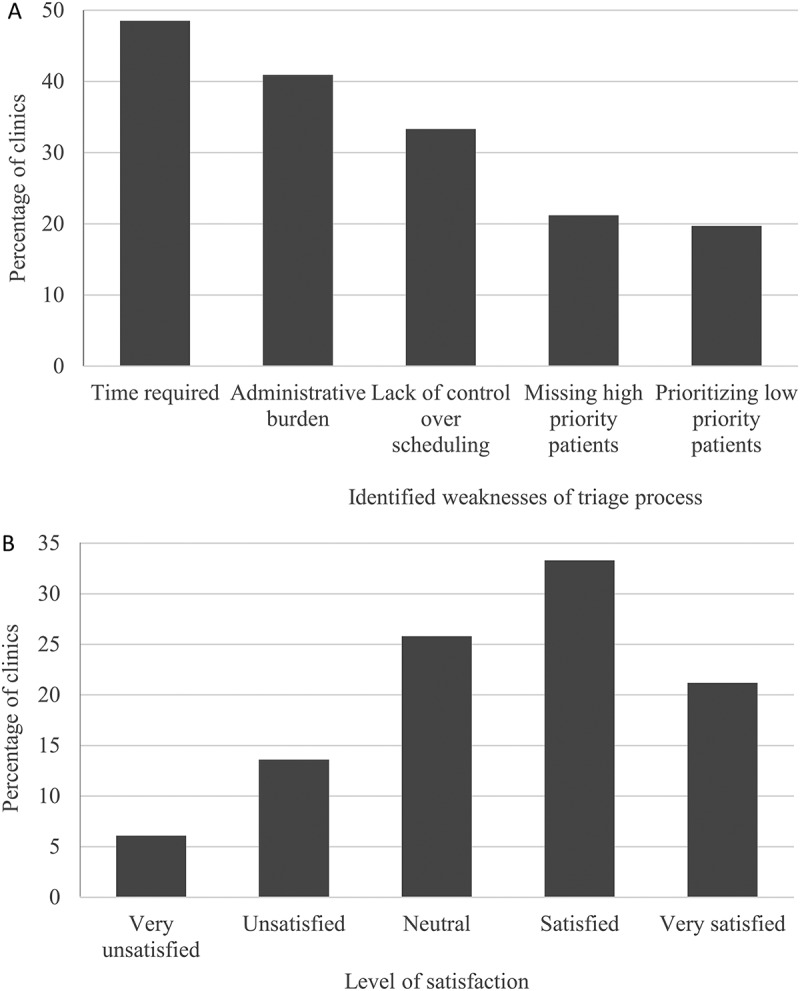
Difficulties with triage processes. (A) Percentage of multidisciplinary pain clinics who have endorsed each item as a weakness of triage process. (B) Level of satisfaction with current triage process.

Approximately half of the multidisciplinary pain clinics (54%) reported being very satisfied or satisfied with triage processes currently in place. The primary identified weaknesses of the triage processes include time requirement, administrative burden, lack of control over scheduling, missing high-priority patients, and prioritizing low-priority patients (Figure 5). In addition, some clinics identified referral volumes, lack of time/staff to triage, and lack of evidence-based practice for optimizing triage as additional weaknesses to triage process (Table 2). There were no significant correlations between level of satisfaction with the triage process and number of new referrals per year, number of new patients seen per year, average wait time or clinic type (private vs. public; all *P* > 0.05).

Results of the binary regression analysis showed that using a structured referral template was a significant predictor of satisfaction with the triage (*B* = 1.22; Wald test = 4.94; *df* = 1; *P* = 0.026).

## Discussion

The study aim was to appraise existing triage systems across multidisciplinary pain clinics worldwide. This information could serve as a primary step toward the development of evidence-based triage guidelines that would minimize waitlist and optimize treatment services.

Results showed that (1) more than one quarter of publicly funded clinics had an average wait time of 6 months or longer; in contrast, all privately funded clinics reported an average wait time shorter than 6 months; (2) triage processes are complex (using eight or nine criteria) for the majority of the clinics studied; a minority of the clinics (6%) solely used the time of referral as the criterion for triage; (3) pain characteristics, psychosocial status, and referral dates are the most often used triage criteria; and (4) though triage processes are satisfactory for just over half of respondents from sampled clinics, time requirements, administrative burden, scheduling barriers, and prioritization shortcomings were identified by many clinics as barriers to their triage process.

### Number of referral, new patients, and wait time

Results from this study are consistent with other research indicating that approximately half of multidisciplinary pain clinics have a wait time averaging 6 months or less.^15^ The present study showed important variations in terms of clinic volume (number of referrals and new patients seen), and this was significantly associated with average wait time. However, clinic volume accounted for less than 20% of the variance in average wait time, suggesting that other factors, such as administrative and availability of resources, could in part explain the variance in wait time.

Interestingly, clinics’ satisfaction with the triage process was not significantly associated with clinic volume or average wait time. Given that the primary aim of triage is to identify priority patients and reduce wait time for those patients, one could expect that clinics with longer wait times would express greater dissatisfaction with the triage process, yet this was not the case in the present study. A recent study found a negative association between wait time and patient satisfaction among patients with chronic non-cancer pain.^25^ No research could be found on the association between clinician satisfaction and average wait time to access services. It would be interesting for future studies to identify the best predictors of clinician satisfaction with the triage process in order to help identify elements essential to develop best practice guidelines for triage strategies.

### Triage processes

Approximately half of clinics were satisfied with current triage processes in place. For those that were dissatisfied with triage process, several weaknesses were identified, including missing high-priority patients and prioritizing nonurgent patients. Though strictly following established rules of triage would optimize this process, the issue of triage failure remains highly dependent on the quality of information gathered as part of the triage process. For example, it is possible that information gathered as part of the initial patient interview would change the priority status of the patient retrospectively; if such information were available at the time of triage, the assigned priority would have been different. The lack, or inadequacy, of structured referral templates might increase the risk of triage failure. It is possible that by implementing an evidence-based standard triage process such failures would be minimized. One way to minimize such misidentification would be to gather comprehensive information about patients for the purpose of triaging by using a structured referral template or relying on patient-administered clinical questionnaires. In fact, results from the present study demonstrated that using a structured referral template is associated with increased satisfaction with the triage process. Similarly, some countries have developed a classification system of chronic pain patients (mild, moderate, or severe). The higher the severity rating, the more likely these patients will require extensive treatment and the less likely they are to respond to interventions. These questionnaires have generally moderate to good psychometric properties.^26,27^ Together, these findings are important in that future triage guidelines might consider designing a standard structured referral template that would contain key elements necessary to triage decision making. Though not directly assessed in this study, it will be important to identify clinical and administrative barriers to the implementation of such a structured referral template leading to decisions regarding patient triage. For example, it is possible that private clinics are under a certain amount of pressure to rapidly see patients to avoid losing the patient. Alternatively, some clinics might be facing legislative requirements that are not compatible with a refined triage process dependent on structured referral templates.

A triage process could also be used to identify patients who might not require multidisciplinary care and who would be scheduled for a one-time assessment with recommendations given to the referring physician. This approach has been found to benefit one quarter of patients according to physician feedback.^21^

Similar to other health care systems, including the emergency department, access to chronic pain services is limited by the finite clinical resources available. Therefore, using a triage system is essential to ensure fairness to patients and for optimizing resources. Unlike pain clinics, triage research of emergency departments is much more advanced with the adoption of standard triage scales in many countries (Australian Triage Scale, Manchester Triage Scale in the United Kingdom, and Canadian Triage and Acuity Scale) and the development of international standards.^28^ The current study has demonstrated the need for such standards that would facilitate identification of urgent cases, reduce administrative burden, facilitate patient flow, and improve outcomes. These standards would need to first establish an empirically based triage prioritization recommendation (e.g., optimal window of interventions for each specific pain etiology and potential treatment response when this time frame is respected) and subsequently validate triage algorithms that would assign priority codes to each patient.

## Limitations

This study provided informative data on existing triage processes in five different countries. Nonetheless, there are some limitations to the survey. First, the survey was only available in English and, as such, was limited to English-speaking countries or countries where English is easily understood. The survey was also limited to countries with a health care system structure similar to Canada’s. This was done in order to optimize comparability of the results. Second, survey dissemination was primarily done through national and international pain associations. It is possible that higher response rates would have been obtained by compiling a list of all pain clinics in the targeted countries and directly approaching these clinics. Due to feasibility and resource availability, this recruitment method was not implemented. From available lists of multidisciplinary pain clinics, response rates ranged from 5.7% in Canada (11/192 clinics^29^) to 40.7% in Australia (44/108 clinics^30^). Third, satisfaction with triage was used to evaluate adequacy of current triage procedures. Though informative, it is also sensitive to specific clinic characteristics and resource availabilities. It will be important for future research examining optimal triage procedures to measure sensitivity and specificity of triage prioritization based on identified outcomes. Lastly, given the differences in health care systems across countries, it is difficult to identify the best triage systems (based on reported triage criteria, wait time, and referral volume) and as such the study was limited to providing a descriptive overview of existing systems.

## Conclusions

The vast majority of surveyed multidisciplinary pain clinics use a triage system, and these systems rely predominantly on pain etiology, type of pain, referral date, psychosocial status, and pain level to prioritize patients. Many difficulties with these systems have been identified, including time requirements, administrative burden, and lack of control over scheduling. Level of dissatisfaction with current triage processes was also high. This study identified a need for the elaboration of best practice clinical guidelines for triage processes including the use of a structured referral template that contains the information necessary for effective triage decision making. Examination of efficiency of the different triage systems currently in place as well as identification of common triage goals and outcomes (e.g., common agreement on type of pain etiology to prioritize) will be the next steps toward the establishment of such evidence-based guidelines.
